# High cognitive load enhances the susceptibility to non-speech audiovisual illusions

**DOI:** 10.1038/s41598-018-30007-6

**Published:** 2018-08-01

**Authors:** Georgios Michail, Julian Keil

**Affiliations:** 1 Department of Psychiatry and Psychotherapy, Multisensory Integration Lab, Charité Universitätsmedizin Berlin, Berlin, Germany; 2 Biological Psychology, Christian-Albrechts-University Kiel, Kiel, Germany

## Abstract

The role of attentional processes in the integration of input from different sensory modalities is complex and multifaceted. Importantly, little is known about how simple, non-linguistic stimuli are integrated when the resources available for sensory processing are exhausted. We studied this question by examining multisensory integration under conditions of limited endogenous attentional resources. Multisensory integration was assessed through the sound-induced flash illusion (SIFI), in which a flash presented simultaneously with two short auditory beeps is often perceived as two flashes, while cognitive load was manipulated using an n-back task. A one-way repeated measures ANOVA revealed that increased cognitive demands had a significant effect on the perception of the illusion while post-hoc tests showed that participants’ illusion perception was increased when attentional resources were limited. Additional analysis demonstrated that this effect was not related to a response bias. These findings provide evidence that the integration of non-speech, audiovisual stimuli is enhanced under reduced attentional resources and it therefore supports the notion that top-down attentional control plays an essential role in multisensory integration.

## Introduction

When one tries to localize a singing bird flitting between the branches of a tree with luxuriant foliage, the combination of auditory and visual input information – as compared to using only auditory or visual information – will probably increase the accuracy and speed of the localisation process. Navigating in an uncertain world, abundant in multisensory objects, requires the constant combination of sensory cues across different modalities, a process known as multisensory integration^1^ (MSI). Indeed, a large body of animal and human studies suggest that sensory processing and discrimination is sharpened when multisensory information is provided (animal studies:^2,3^, human studies:^4–8^). Recent studies have shown that multisensory perception is not a hardwired routine, but on the contrary, it is influenced by a wide range of neurophysiological processes such as the power and phase of ongoing oscillations and several cognitive factors such as the level of attention and expectations^9^.

Regarding specifically the role of attention during the integration of multisensory information, studies point towards a complex relationship that unfolds at different levels of sensory processing^10,11^. The exact nature of the MSI-attention interplay is largely determined by the involved sensory modalities that can, for instance, have different spatio-temporal detection accuracies^7,12–15^. Moreover, the interaction between MSI and attention is influenced by the specific characteristics of the stimuli such as the stimulus intensity (e.g., near- vs. supra-threshold stimuli^16^) and complexity (e.g., speech vs. simple audio-visual stimuli^17,18^. Additionally, this interaction is influenced by the conditions of sensory stimulation such as the noisiness of the background or task-specific requirements narrowing the perceiver’s focus on one modality or a specific stimulus feature^11^. The degree of association between the unimodal components of a multisensory signal was also proposed as a factor that determines the extent of attentional effects on multisensory integration^19^. Based on this proposal, the integration of strongly associated unimodal signals (e.g., audio-visual input during natural speech) is less likely to be affected by attentional factors compared to unimodal signals that are weakly associated due to spatial, temporal or semantic incongruencies.

An ongoing debate revolves around the question whether and under which conditions the binding of multisensory stimuli occurs automatically (or pre-attentively) or is influenced by top-down attentional control^20^. Whereas some studies support that MSI is modulated by attention^21–23^, others provide evidence that it can also take place in a pre-attentive, automatic way^24–27^. It seems, that the influence that attention exerts on MSI is defined by the combined attentional effect of the bottom-up signalling and the endogenous attentional mechanisms^11^. The relative contribution of the two factors is to a large extent situation-dependent, and thus difficult to define precisely.

One approach to tackle this question is the use of a dual task paradigm in which one task is used to modulate the levels of endogenous resources available for the secondary task. Using this approach, Santangelo and Spence showed that under high perceptual load only audiovisual – and not auditory or visual – cues managed to capture visuo-spatial attention, indicating the effectiveness of multisensory stimulation in orienting spatial attention under high perceptual load^6^.

The small number of studies that employed this dual task design to directly explore the effect of limited attentional resources on the integration of multisensory stimuli provided contradicting results^27–29^. Among these, two recent studies reported that audio-visual speech integration, as indexed by the McGurk effect – where a speech sound presented together with incongruent lip movement is perceived as a different, illusory, speech sound – was reduced under high attentional load^28,29^. On the contrary, Zimmer and Macaluso found that visuo-tactile spatial integration was insensitive to load manipulations of working memory and visuo-spatial attention^27^. Given the contradicting findings of these studies as well as their focus on different aspects of MSI (audio-visual speech perception^28,29^; visuo-tactile spatial integration^27^), it appears that several aspects related to the effect of load on MSI have not been sufficiently studied. Importantly, it remains an open question whether the finding of reduced audio-visual speech integration under high attentional load^28,29^ is relevant for the binding of simple, non-linguistic, audio-visual information. The increased reliance on sound under load might be to a large extent a speech-specific characteristic as we naturally rely more on sound than on vision for speech recognition. Furthermore, audio-visual speech is suggested to be a specific type of multisensory integration^30,31^. Another limitation of speech as a paradigm for the investigation of MSI–attention interactions is that one cannot exclude participants’ strategic use of pre-existing associations related to the semantic content of the stimuli^11,32^.

**Figure 1:**
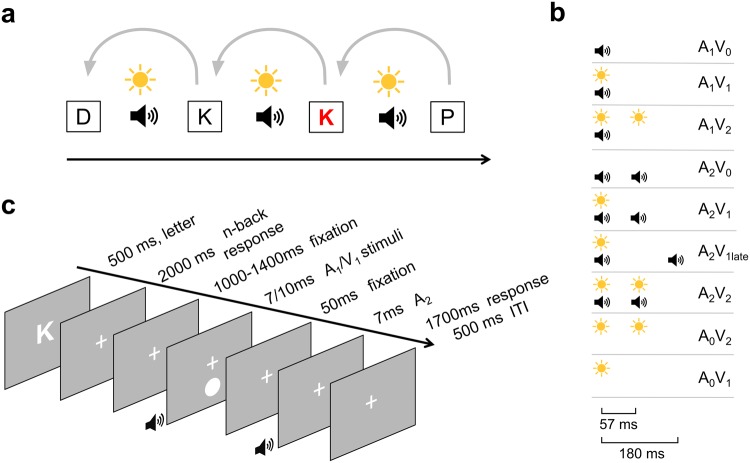
Schematic Illustration of experimental paradigm and material. (**a**) Representation of the dual task design for the 1-back condition. Participants were presented a letter and had to indicate if it matched the letter in the n-th previous trial. After the letter presentation the SIFI audiovisual stimuli were presented and participants had to report the number of perceived flashes. (**b**) The 9 audio-visual stimuli combinations that were used in the experiment. The stimulus onset asynchrony (SOA) was 57 ms for all combinations except the control condition A_2_V_1late_ (180 ms SOA). (**c**) Illustration of a single critical A_2_V_1_ trial depicting the different parts of the trial, the intervals in between these parts, and the duration of the stimuli.

The aim of the present study was to assess the effect of varying levels of cognitive load on the integration of simple, non-linguistic, audio-visual stimuli. We employed a robust audio-visual illusion paradigm, the so-called “Sound-Induced Flash Illusion” (SIFI) in which a single flash presented simultaneously with two auditory beeps is sometimes perceived as two flashes^33^ (see Fig. 1). In the SIFI, the degree of audio-visual integration is assessed in terms of the illusion rate. We used an additional, orthogonal n-back task to manipulate the attentional resources that were available for the processing of multisensory input. We assume that increased working memory load requires additional resources, thus limiting resources available for other cognitive processes. This assumption is based on the influential model of working memory proposed by Baddeley and Hitch^34^ (and updated later by Baddeley^35^) that involves the interaction of attentional control (performed by the central executive) with the maintenance of information in the storage systems (phonological loop and visuo-spatial sketchpad) and the episodic buffer. A growing body of behavioural and neuroscientific studies suggests that attention and working memory are functionally inter-twined and show extensive neuroanatomical overlap, involving fronto-parietal brain regions^36–40^. Importantly, Gazzaley and Nobre^41^, taking into account neurophysiological evidence, propose that the top-down modulatory mechanism underlying selective attention processes during perceptual processing is also engaged during the different stages of working memory – encoding, maintenance, and memory retrieval. Within this framework, we expect that an increase of working memory load due to the n-back task would present increased demands on cognitive resources thus limiting the resources available to attentional mechanisms for the processing of the upcoming audiovisual stimuli. Interestingly, an fMRI study showed that visuo-tactile spatial integration was independent from both working memory and attentional resources^27^. However, the finding of another study showing that audiovisual cues, as compared to unimodal, were more efficient in biasing access information in visuo-spatial working memory, indicates that multisensory integration can affect working memory performance^42^.

To produce the SIFI illusion, participants were presented with a single flash paired with two auditory beeps in rapid succession. Eight other flash-beep combinations (of 0, 1 and 2 flashes and beeps) were used to control for perception of auditory-only, visual-only, and congruent audiovisual stimuli as well as for a response bias in the SIFI illusion (by using the same design as in illusory trials but with increased inter-beep interval) and an alternative illusory phenomenon called “Fusion” illusion – i.e., the illusory perception of a single flash when two flashes are paired with a single beep^43^. In all conditions, participants were asked to report the perceived number of flashes. The cognitive resources available for the SIFI task were manipulated by an n-back task performed prior to the SIFI task, in which participants were asked to indicate if the letter presented in the current trial matched the one presented *n* trials before. Varying the *n* (highest was 2), enabled us to examine the effect that different degrees of cognitive load have on the multisensory integration of simple audio-visual stimuli, as indexed by the SIFI illusion rate. An altered susceptibility to audiovisual illusions under high cognitive load and thus under reduced attentional resources would suggest a regulatory role of attention in audio-visual integration. Additionally, the direction of a possible effect – larger or smaller susceptibility – would provide further insights about the modulatory effect that attention exerts on multisensory integration. Based on the finding of reduced audio-visual speech integration (McGurk effect) under high attentional demands^28^, one could expect a similar decline in the SIFI illusion rate under high cognitive load. However, whether this assumption is valid needs to be tested given the differences between the McGurk effect and the SIFI illusion in terms of the nature of the audio-visual stimuli (speech vs. non-speech) and the reported modality (sound vs. vision).

## Results

### N-back

The n-back task performance was assessed in terms of accuracy and reaction times (RTs). The mean RTs and accuracy, for all the different working memory (WM) load levels are displayed in Table 1. Data are provided throughout the text as mean and, in square brackets, standard deviation (SD), unless otherwise noted.

**Table 1:** Mean (SD) reaction times and accuracy for the different n-back levels.

n-back level	0-back	1-back	2-back
Accuracy (%)	92.41 (19.18)	92.71 (5.01)	85.26 (11.91)
Reaction time (s)	0.40 (0.03)	0.55 (0.04)	0.70 (0.06)

Our analysis revealed that WM load had a significant effect on accuracy (Friedman’s test, *p* = 0.002, *χ*^2^ = 12.79, df = 2). Wilcoxon signed-rank post-hoc tests revealed that the subjects displayed significantly lower accuracy in 2-back compared to 0-back trials (85.26 [11.91] % for 2-back and 92.41 [19.18] % for 0-back, Z = −2.44; *p* = 0.045, *r* = 0.35) and 1-back trials (92.71 [5.01] % for 1-back, Z = −2.43; *p* = 0.045, *r* = 0.35).

Moreover, we found that reaction times, associated with correct responses to targets, were affected by WM load (F_(2, 30)_ = 25.89; *p* < 0.001). Post-hoc paired-samples *t*-tests showed that subjects displayed significantly longer RTs in the 2-back trials compared to both 1-back (mean RT was 0.70 [0.06] s for 2-back and 0.55 [0.04] s for 1-back, t(15) = 3.47, *p* = 0.003, BF = 13.45) and 0-back trials (mean RT was 0.40 [0.03] s for 0-back, t(15) = 6.12, *p* < 0.001, BF = 1220.57). Also, RTs in 1-back trials were longer than in 0-back (t(15) = 4.98, *p* < 0.001, BF = 184.46).

Overall, our data show that with increasing level of difficulty the subjects display lower accuracy and slower RTs. Thus, these results demonstrate the efficacy of the n-back task to modulate the working memory load and limit attentional resources.

### Sound-induced Flash illusion

The performance in the SIFI task was assessed in terms of the number of perceived flashes and the reaction times. The mean (SD) percentage of the analysed flash responses in all n-back levels and combinations of audiovisual stimuli is reported along with a summary of the results of the statistical analyses in Table 2. The same information for the RTs is reported in Table 3.

**Table 2:** Mean (SD) percentage of the analysed flash responses and a summary of the results of statistical analyses (ANOVA - Friedman’s test) regarding the effect of WM on the perception of audiovisual stimuli in the SIFI task.

AV condition	Flash Response	Mean (SD) percentage of analysed flash responses (%)				Statistical Analysis			
		no-back	0-back	1-back	2-back	*ANOVA*			
						F	df_hypothesis_	df_error_	*p*
A_2_V_1_*	2	41.46 (27.24)	49.38 (28.00)	51.88 (29.89)	57.08 (27.32)	4.56	3	45	0.007
						*Friedman’s Test*			
						χ^2^	df		*p*
A_0_V_1_	1	92.2 (8.86)	93.23 (5.46)	93.23 (8.18)	91.67 (12.17)	0.18	3		0.98
A_0_V_2_	2	92.71 (11.33)	89.06 (14.18)	93.23 (7.59)	91.15 (11.97)	2.07	3		0.56
A_1_V_0_	0	98.44 (4.53)	94.79 (8.54)	96.35 (5.24)	94.27 (7.89)	7.34	3		0.06
A_1_V_1_	1	98.44 (3.36)	96.88 (5.99)	96.35 (5.24)	97.40 (5.87)	2.15	3		0.54
A_1_V_2_*	1	26.04 (28.69)	16.15 (25.18)	19.27 (24.67)	16.67 (23.77)	6.05	3		0.11
A_2_V_0_	0	97.40 (3.99)	95.83 (6.09)	92.71 (11.33)	93.23 (11.06)	2.55	3		0.47
A_2_V_2_	2	95.31 (6.78)	96.88 (6.72)	99.48 (2.08)	98.96 (2.85)	8.40	3		0.04
A_2_V_1late_*	2	17.19 (23.27)	25.52 (22.25)	28.13 (29.48)	25.52 (22.04)	2.84	3		0.42

*In the incongruent stimuli combinations, the analysed flash response was the “illusory” response (e.g., the 2-flash response in A_2_V_1_). In all the other control stimuli combinations, the analysis was performed on the percentage of correct responses.

**Table 3:** Mean (SD) RTs and a summary of the results of statistical analyses (ANOVA - Friedman’s test) regarding the effect of WM on the RTs after the presentation of AV stimuli in the SIFI task.

AV condition	Mean (SD) Reaction Time (s)				Statistical Analysis			
	no-back	0-back	1-back	2-back	*ANOVA*			
					F	df_hypothesis_	df_error_	*p*
A_0_V_1_	0.624 (0.14)	0.683 (0.10)	0.693 (0.10)	0.714 (0.14)	4.86	3	45	0.005
A_0_V_2_	0.617 (0.15)	0.719 (0.11)	0.675 (0.12)	0.708 (0.14)	7.28	3	45	<0.001
A_1_V_0_	0.668 (0.14)	0.781 (0.16)	0.814 (0.19)	0.776 (0.15)	12.87	3	45	<0.001
A_1_V_2_	0.670 (0.18)	0.733 (0.13)	0.728 (0.16)	0.737 (0.17)	2.80	2.15	32.24	0.072
A_2_V_1_	0.725 (0.18)	0.761 (0.13)	0.787 (0.13)	0.777 (0.14)	2.51	2.30	34.54	0.089
A_2_V_1late_	0.743 (0.14)	0.853 (0.17)	0.839 (0.17)	0.825 (0.12)	4.57	3	45	0.007
					*Friedman’s Test*			
					*χ* ^2^	df		*p*
A_1_V_1_	0.568 (0.15)	0.643 (0.14)	0.654 (0.13)	0.663 (0.13)	9.38	3		0.025
A_2_V_0_	0.690 (0.17)	0.822 (0.17)	0.808 (0.19)	0.804 (0.17)	20.63	3		<0.001
A_2_V_2_	0.594 (0.18)	0.652 (0.14)	0.682 (0.15)	0.687 (0.15)	16.73	3		0.001

### Working memory load manipulation affects SIFI perception

**Figure 2:**
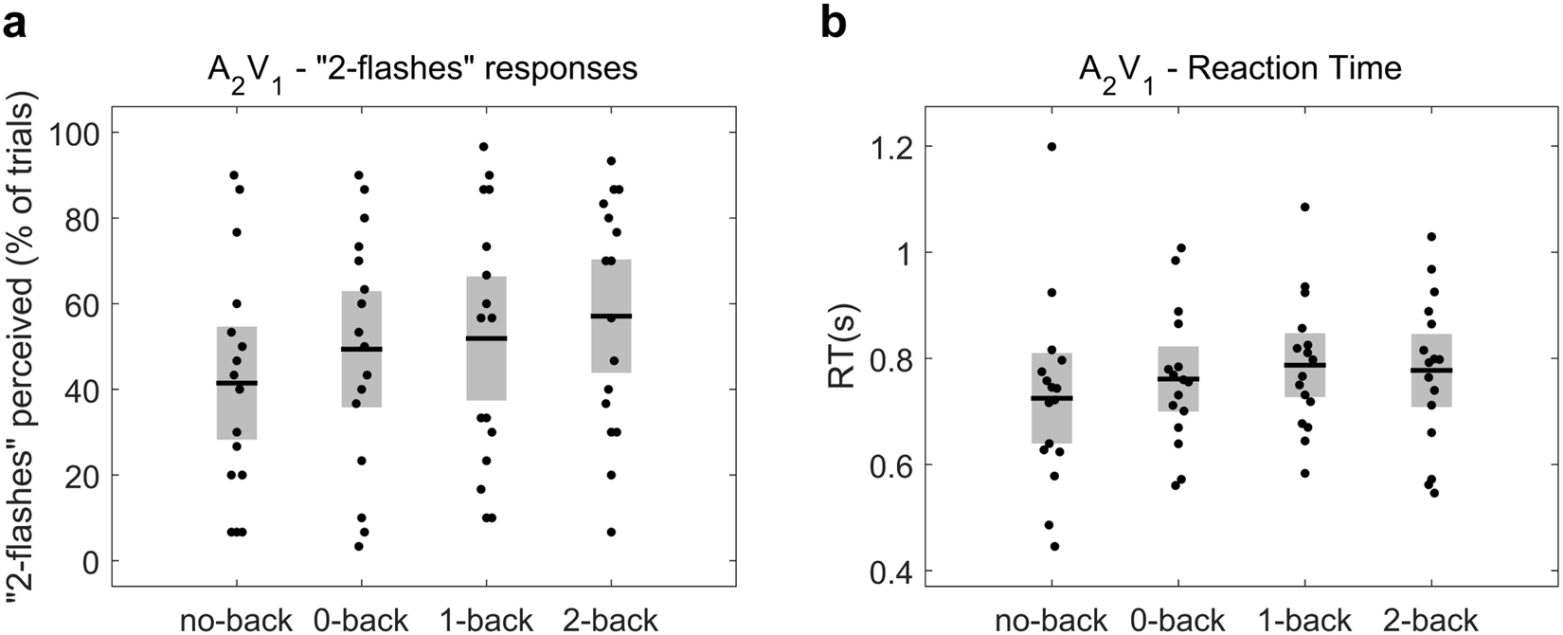
Increased illusion rate under high working memory load in critical A_2_V_1_ trials. (**a**) The percentage “2-flashes” responses in A_2_V_1_ trials – i.e., illusion rate – for the different working memory levels. A one-way ANOVA revealed that working memory load had a significant effect on the illusion perception. Post-hoc comparisons showed that illusion perception in 2-back was significantly higher compared to no-back and relatively higher compared to 0-back (**b**) The RTs for reporting the perceived flashes number in A_2_V_1_ trials for all the working memory load levels. No significant effect of load on the RTs was found. Horizontal black lines denote the mean and grey bars the standard error of the mean.

To assess the effect of WM load on the perception of the sound-induced flash illusion, we analysed the differences in the illusion rate i.e. the percentage of “2-flashes” responses in the critical trials (A_2_V_1_) – between the different WM load levels. One-way repeated-measures ANOVA showed that WM load had a significant effect on the illusion rates (F_(3, 45)_ = 4.56, *p* = 0.007), in the direction of increasing illusion rates with increasing WM load as can be seen in Fig. 2a. Post-hoc comparisons revealed a significantly larger illusion perception in 2-back trials compared to no-back (mean illusion rate was 57.08 [27.32] % for 2-back and 41.46 [27.24] % for no-back, t(15) = 3.12, p = 0.043, BF = 7.27). Additionally, the Bayes Factors provided some evidence that the illusion perception was stronger in 2-back trials compared 0-back (mean illusion rate was 49.38 [28.00] % for 0-back, t(15) = 2.63*, p* = 0.095, BF = 3.25), stronger in 1-back trials compared to no-back (mean illusion rate was 51.88 [29.89] % for 1-back, t(15) = 1.96, *p* = 0.23, BF = 1.17) and stronger in 0-back compared to no-back (t(15) = 2.06, *p = *0.23, BF = 1.35). We repeated the same one-way repeated measures ANOVA using “normalised” illusion rates (obtained after dividing the percentage of 2-flashes responses in A_2_V_1_ by A_0_V_2_) and found a significant main effect of WM load (F_(2.25, 33.73)_ = 3.89; *p* = 0.026), indicating that the observed effect is not affected by possible individual biases in the perception of two flashes. These findings indicate that the illusory perception of two-flashes when a single flash is presented together with two auditory stimuli is enhanced when the attentional resources are limited.

In addition, we also explored whether WM load affected the magnitude of the SIFI illusion perception in the group of excluded subjects (N = 14). We found that in this highly heterogeneous group there was no significant effect of WM load on the perception of the SIFI illusion (i.e., percentage of 2-flash responses in the A2V1 trials; Friedman’s test, p = 0.15, χ2 = 5.38, df = 3). A separate analysis for each excluded subgroup was not possible due to the small number of subjects in the subgroups.

**Figure 3:**
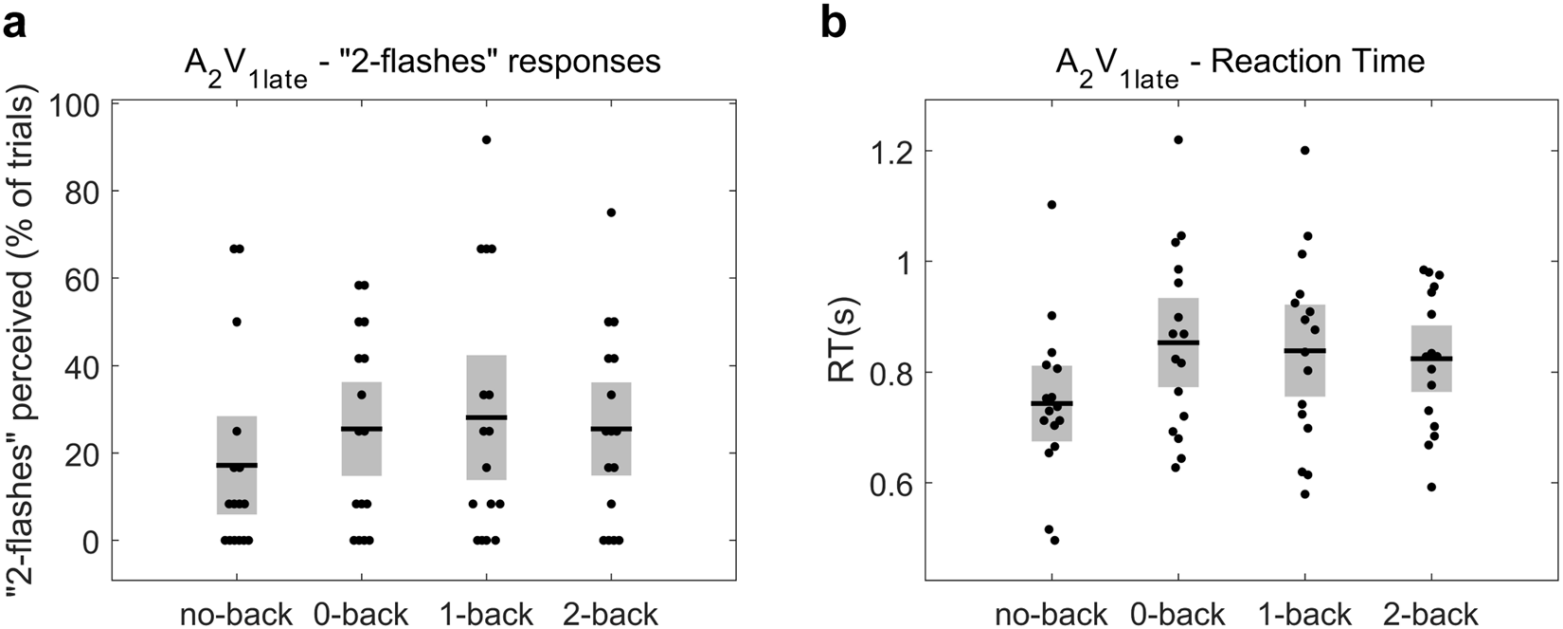
Working memory load doesn’t affect illusion rates in control A_2_V_1late_ trials. (**a**) The percentage “2-flashes” responses in A_2_V_1late_ trials for the different working memory levels. The analysis revealed that working memory load had no significant effect on the illusion perception. (**b**) The RTs for reporting the perceived flashes number in A_2_V_1late_ trials for all the working memory load levels. Horizontal black lines denote the mean and grey bars the standard error of the mean.

To examine whether the effect of WM load on the illusion perception was related to a response bias – as compared to an effect on perceptual mechanisms – we performed a similar analysis on the control condition A_2_V_1late_ in which the second auditory stimulus was presented with increased latency compared to the A_2_V_1_ trials (see Fig. 1b). Figure 3 represents participants’ illusion rate and the RTs for A_2_V_1late_ trials. If the participants’ illusion rates (“2-flash” responses) in the critical A_2_V_1_ trials were based on a reflective response to the number of presented auditory stimuli, we would expect to find an effect of WM load on the “2-flash” responses for the A_2_V_1late_, similar to the effect found in A_2_V_1._ Our analysis revealed that WM load had no significant effect on the percentage “2-flash” responses in the A_2_V_1tlate_ trials (Friedman’s test, *p* = 0.42, *χ*^2^ = 2.84, df = 3). This result indicates that the observed effect of WM load on the illusion perception (in A_2_V_1_) was not related to a response bias but was primarily due to changes on perceptual mechanisms linked to varying levels of working memory load.

**Figure 4:**
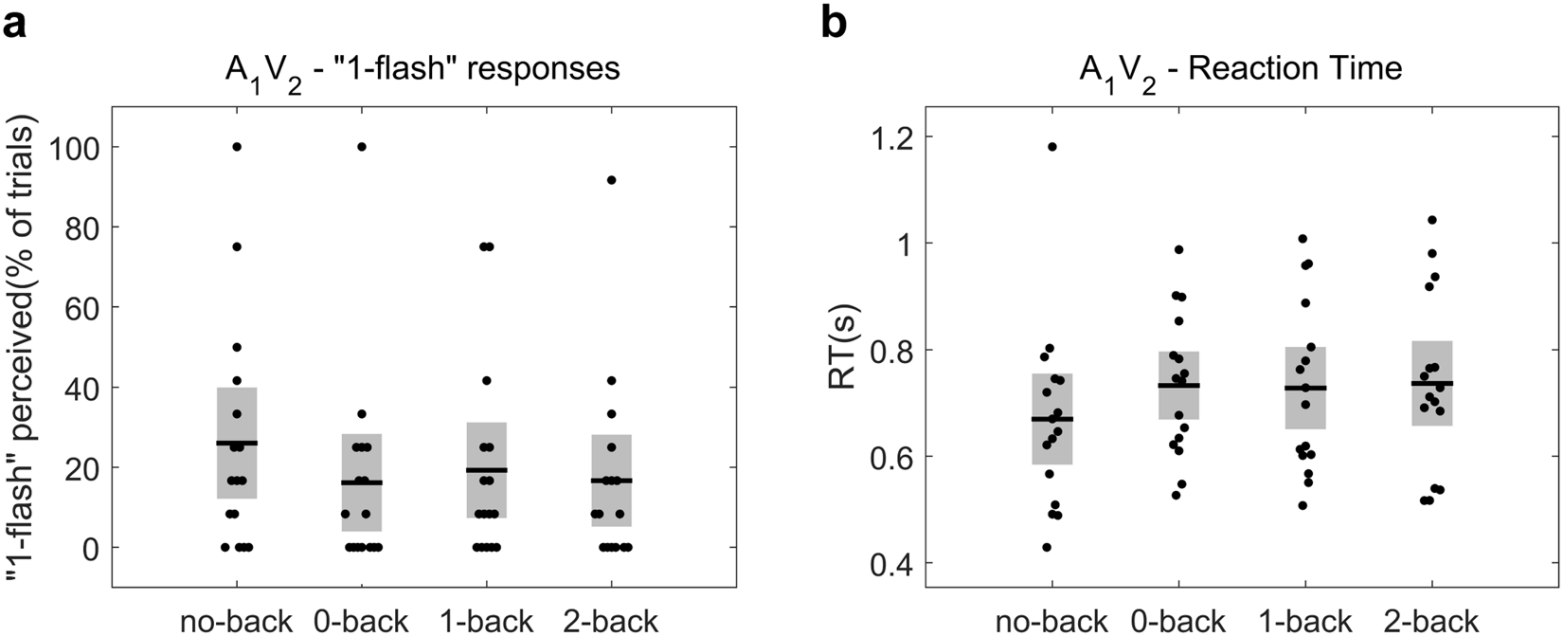
No influence of working memory load on the “Fusion” illusory percept in A_1_V_2_ trials. (**a**) The percentage “1-flash” responses in A_1_V_2_ trials – indexing the strength of the “Fusion” percept – for the different working memory levels. Our analysis demonstrated no significant effect of working memory load on the “Fusion” perception. (**b**) The RTs for reporting the perceived flash number in A_1_V_2_ trials for all the working memory load levels. No significant effect of load on the RTs was found. Horizontal black lines denote the mean and grey bars the standard error of the mean.

Additionally, a similar analysis for the A_1_V_2_ condition – associated with the “Fusion” illusion in which two flashes are ‘fused’ and perceived as one –, revealed that WM load had no significant effect on the percentage “1-flash” responses (Friedman’s test, *p* = 0.11, *χ*^2^ = 6.05, df = 3; Fig. 4). This finding suggests that the effect that WM load has on audio-visual perception might be specifically related to the perceptual mechanisms underlying the SIFI – “Fission” –illusion (one flash perceived as two) that differ from the processes underlying the “Fusion” illusion^43^. The effect of WM on the percentage of correct responses in all the other control conditions was also investigated. The results of the statistical analyses are reported in Table 2. There was no significant effect of WM load on the correct responses in any of the control conditions except the A_2_V_2_ (Friedman’s test, *p* = 0.04, *χ*^2^ = 8.40, df = 3). However, the percentage of “2-flashes” responses was not statistically different between the different n-back levels (*p* > 0.05 in all post-hoc pairwise comparisons). Therefore, and given the small number of A_2_V_2_ trials (12 per n-back level) – that could lead to inflated percentage differences –, this result should be interpreted with caution and needs to be verified in further studies using larger trial numbers.

### Working memory load manipulation and reaction times

At the next step, we first examined whether WM load affected the reaction times of participants when they reported the perceived flashes number, for the audio-visual combinations A_2_V_1_, A_2_V_1late_ and A_1_V_2_. We found that WM load had no significant effect on the RTs for the critical A_2_V_1_ trials (F_(2.30, 34.54)_ = 2.51, *p* = 0.089; Fig. 2b), as well as on the RTs for the A_1_V_2_ trials (F_(2.15, 32.24)_ = 2.80, *p* = 0.072; Fig. 3b). In contrast, WM load had a significant effect on the RTs for the A_2_V_1late_ trials (F_(3, 45)_ = 4.57, *p* = 0.007; Fig. 4b). Post-hoc tests show that in A_2_V_1late_ trials the RTs were significantly slower in 2-back compared to no-back, (mean RT was 0.825 [0.12] s for 2-back vs. 0.743 [0.14] s for no-back, t(15) = 3.13, *p* = 0.041, BF = 7.48) and provide some evidence for slower RTs in 1-back compared to no-back (mean RT for 1-back was 0.839 [0.17] s, t(15) = 2.59, *p* = 0.082, BF = 3.05) and in 0-back compared to no-back (mean RT for 0-back was 0.853 [0.17] s, t(15) = 2.89, *p* = 0.056, BF = 4.98).

Interestingly, a similar analysis performed in the other control conditions (A_0_V_1_, A_0_V_2_, A_1_V_0_, A_1_V_1_, A_2_V_0_, A_2_V_2_) showed that WM had a significant effect on RTs in all conditions (mean (SD) RT and summary of the results of statistical analyses can be found in Table 3), in the direction of larger RTs with increasing WM load (e.g., in A_0_V_2,_ mean RT was 0.708 [0.14] s for 2-back and 0.617 [0.15] s for no-back, t(15) = 3.48, *p* = 0.017, BF = 13.64). Therefore, these results show that increased WM load resulted in a general slowing of the response after the presentation of audio-visual stimuli except in trials with incongruent audio-visual stimuli (A_2_V_1_, A_1_V_2_). This might be related to the fact that in these two conditions the RT was shaped not only by WM load but also by the incongruency between the auditory and visual stimuli, the degree of which was – contrary to WM load – unvarying across n-back levels.

## Discussion

In the present study, we analysed the audiovisual integration of simple, non-linguistic stimuli as indexed by the strength of the SIFI effect, under different levels of working memory load. We used an n-back task to manipulate the amount of cognitive resources that were available for the processing of the SIFI stimuli. Our main result was that participants displayed enhanced susceptibility to the SIFI under high working memory load. The absence of such effect in the control condition, in which the inter-beep interval was increased, argues against the possibility of our main result being associated with a response bias. Our finding provides strong evidence that audiovisual integration can be modulated by the amount of available cognitive resources and it therefore argues against a pre-attentive account of multisensory integration.

Previous studies, using a range of multisensory tasks, have demonstrated that cross-modal binding can be immune^24,25^, but it can also be sensitive^21,44,45^ to spatial attention manipulations. Also, it’s unclear whether MSI is affected^46^ or not^47^ by modality-specific attention. Although these studies examined particular aspects of the role of attention during the integration of multisensory signals (spatial attention, modality-specific attention), the present study addressed a critically different question. We manipulated endogenous attention using a secondary, orthogonal, n-back task and asked whether multisensory integration is affected under conditions of increasingly limited attentional resources.

The present study is the first, to our knowledge, to demonstrate that limiting the available attentional resources prior to the stimulus delivery enhances the integration of simple, non-linguistic, audiovisual signals. Some previous studies using a similar approach reported that visuo-tactile spatial integration^27^ and the integration of emotional cues in songs^48^ are not affected by increased attentional demands. These studies are not necessarily in conflict with our findings, because their focus and design are in several aspects different to the present study. Zimmer and Macaluso^27^ investigated spatial integration of visual-tactile cues, whereas we focus on the temporal integration of audiovisual stimuli. Also, in Thompson *et al*.^48^, the complexity of the material (songs) and the level of integration process (emotional cue binding) are quite higher compared to the corresponding features of the present study (low-level binding of simple audiovisual stimuli). There is evidence that multisensory events involving different combinations of sensory modalities or stimulus characteristics activate different brain networks^49^, and that the nature of a particular multisensory event affects its susceptibility to attentional manipulations^11,16^.

Yet, previous studies on audiovisual temporal integration showed that audiovisual speech perception, as indexed by the McGurk effect, is sensitive to attentional load manipulations^28,29,50^. Interestingly, these studies demonstrated a reduction in the perception of the McGurk effect under high attentional load, a finding that appears to contradict our results. However, the SIFI and McGurk illusions are characterised by distinct temporal integration properties^51^ and such differences can account for the discrepancy (see next paragraph). We also assume that the experimental design of the present study has some advantages over previous studies. First, the use of simple, non-linguistic stimuli in the present study, excludes the strategic use of pre-existing associations to which speech is sensitive^11,32^. Second, the design of the secondary task in the previous McGurk studies^28,50^ involving the presentation of visual or auditory objects (e.g., shapes superimposed on the faces showing the speech gestures) sometimes temporally overlapping with the McGurk stimuli^28^ complicates the interpretation of the results. This is supported by the discrepancy between these studies, in regard to whether the reduction of the McGurk illusion is attributed to the depletion of attentional resources^28^ or to modality-specific attention^50^. Importantly, the attentional manipulation in the present study through an n-back task was temporally separated from the primary task (SIFI). This excludes or limits to a great extent the potential interference of secondary task material with the audiovisual processing of primary task stimuli. As such, our experimental design enables the firm conclusion that the enhanced audiovisual integration was induced by the reduced levels of endogenous attentional resources that were available for the processing of the multisensory input.

A mechanism that could account for the present findings relates to the temporal window of integration (TWI), i.e., the maximum temporal asynchrony between two different sensory events that allows their perceptual binding into a singular percept^52^. Previous work has demonstrated that susceptibility to audio-visual illusions such as the SIFI can be predicted by individual differences in the temporal window of integration^51^. The integration window increases with age^53^ and it can be recalibrated after exposure to asynchronous stimuli^54,55^. Moreover, the TWI can be adaptively adjusted depending on the task demands^56^. Therefore, it can be assumed that increasing the attentional demands in our experiment might have resulted in an adaptive widening of the individual TWI that in turn led to the enhanced binding of the audiovisual input. This mechanism can also explain the discrepancy between our observation of enhanced SIFI perception under load and the decline of the McGurk effect under increased attentional demands, reported by Alsius *et al*.^28,29^. Previous work has shown that larger TWI is associated with increased susceptibility to SIFI and reduced susceptibility to the McGurk effect^51^. Therefore, a widening of the integration window, induced by high cognitive load, could enhance the illusory perception in SIFI but, on the contrary, diminish the susceptibility to the McGurk effect. What neural mechanism could account for this putative effect? Given the role of alpha oscillations in the temporal sequencing of audio-visual signals^57^, and the temporal resolution in visual^58^ and SIFI-type audiovisual perception^59,60^, it can be hypothesized that a modulation of the alpha oscillations induced by varying attentional demands might have mediated the changes in sensory processing that led to the increased illusory percept. Interestingly, Cecere *et al*.^60^ showed that modulating the individual alpha frequency using electrical stimulation resulted in changes in the TWI. Whether varying attentional demands also modulate neural oscillations, and whether this can explain the current findings requires further testing using electrophysiological methods (M/EEG, ECoG).

Our findings may also be explained based on the “attentional load theory”, which postulates that when high-level cognitive processes are loaded, the processing of task-irrelevant information is enhanced^61^. Because in our experimental design auditory stimuli are less relevant than visual – since participants are instructed to report the number of flashes – it’s possible that under limited attentional resources the auditory input gained a larger sensory weight, which resulted in the enhanced illusion rate. Similarly, the “gating-by-inhibition” hypothesis posits that alpha band oscillations optimize stimulus processing by inhibiting task-irrelevant cortical areas^62^. Increased attentional demands might have interfered with this gating mechanism, coincidentally enhanced neural excitability and increased the crossmodal influence^63^.

Taken together, our findings highlight the influence that attention exerts on audiovisual integration and suggest that when attentional resources are depleted, the cross-modal binding of simple, non-linguistic audiovisual signals is enhanced. These results are especially relevant for the understanding of the interplay between attention and multisensory integration because they provide strong evidence against a pre-attentive account of audio-visual temporal integration. Characterizing this interaction at the behavioural level is an essential first step^64^. Further neuroimaging and electrophysiological studies could provide insights about the neural correlates of this interaction and the stage of sensory processing at which attentional effects occur. Further studies could also include lure trials in order to control for the use of familiarity signals during the n-back task^65^. Another interesting question that should be addressed in future investigations is whether there is a different effect of cognitive load on multisensory integration between target and non-target n-back trials. This question couldn’t be addressed in the context of the current study, due to the small number of target A_2_V_1_ trials and the different ratio of target to non-target trials between the different n-back levels. In the current experiment, the use of a fixed inter-beep interval in the critical A_2_V_1_ trials might have resulted in the extremely high and low SIFI illusion rates we observed in some of the excluded subjects. To alleviate this, future studies could adjust the inter-beep interval individually to account for the inter-individual variability in the temporal window of integration^51,66^.

## Methods

### Subjects

Thirty healthy subjects (10 males, mean age = 29.9 years, SD = 7.8, range = 20–56) participated in this study after providing written informed consent. All participants reported normal hearing, normal or corrected-to-normal vision and absence of any neurological condition. The study was conducted in accordance with the Declaration of Helsinki and approved by the ethics committee of the Charité–Universitätsmedizin Berlin.

### Task Design

The subjects performed a dual task paradigm (Fig. 1a) that combined a visual verbal n-back task and the SIFI paradigm adapted from Shams *et al*.^67^. The n-back task was used to present increased demands on working memory (WM) and therefore reduce the available cognitive resources for the processing of the SIFI audio-visual stimuli. The experiment consisted of 12 blocks corresponding to 3 blocks for each of the 4 levels of WM load (no-back, 0-back, 1-back, 2-back). Each experimental block contained 10 critical A_2_V_1_ trials (two auditory and one visual stimulus) and 4 trials for each of the other 8 combinations (see *SIFI audio-visual stimuli*, Fig. 1b). In total, the experiment included for each WM load category, 30 critical A_2_V_1_ trials and 12 trials for all the other combinations. The order of the blocks was randomized across participants. The experiment was conducted in a sound-attenuated chamber using a portable computer (HP Pavilion 17) and lasted for around 43 min, excluding the short breaks between the blocks. Participants performed 10 practice trials for each WM load category, prior to the main part of the experiment. The Psychophysics toolbox^68^ for MATLAB (The Mathworks, Natick, MA, USA) was used for presenting the stimuli and obtaining the responses. Data analyses were performed using MATLAB (The Mathworks, Natick, MA, USA) and SPSS software (SPSS Inc., Chicago, IL, USA).

As illustrated in Fig. 1c, each trial of the 0-, 1- and 2-back blocks started with a letter presented for 500 ms, followed by a window of 2000 ms in which the participants were instructed to indicate if the currently presented letter matched the one presented *n* trials before (1-back and 2-back) or with a predefined letter “X” (0-back). No response was required for non-targets. After a randomized 1000–1400 ms window, participants were presented with a combination of auditory and visual stimuli. In the case of A_2_V_2_ combination, a pair of – temporally aligned – visual and auditory stimuli was presented followed by another similar pair after a time lag of 57 ms. The visual stimulus was presented for 10 ms and the auditory stimulus for 7 ms. The same timing was followed in all the other combinations (see *SIFI audio-visual stimuli*) except the control condition A_2_V_1late_ in which the second auditory stimulus was presented 180 ms after the first auditory stimulus (based on Mishra *et al*.^69^). Directly after the last stimulus, in the response window (1700 ms), the participants indicated the number of perceived flashes (0, 1, 2). The next trial started after an inter-trial interval (ITI) of 500 ms.

The trials of the no-back blocks were structured in the same way, excluding the ‘n-back’-related periods (letter presentation and the subsequent response window). A fixation cross was displayed throughout the entire trial length. Participants responded with the right thumb (number of flashes) or index finger (n-back targets) using a handheld gamepad (Logitech Gamepad F310, Logitech, Lausanne, Switzerland).

### n-back task stimuli

The stimuli for the n-back task were upper case letters presented in white on a neutral grey background, at the centre of the screen. For each block a (pseudo)random sequence of letters was selected from the set of English consonants. To avoid the use of phonemes as a strategy, vowels were excluded^70^. In the 0-back trials, the target was always the letter “X”. To ensure equal difficulty in all the 2-back sequences, we explicitly manipulated the sequences to exclude the occurrence of – potentially confusing – lure trials, that is, trials in which the presented letter is the same with the one presented in the previous trial, but different to the letter presented 2 trials before. In each sequence, 33% of the letters were targets.

### SIFI audio-visual stimuli

Nine stimulus combinations were presented (Fig. 1b), consisting of 0, 1 or 2 auditory (A) stimuli combined with either 0, 1 or 2 visual (V) stimuli (A_0_V_1_, A_0_V_2_, A_1_V_0_, A_1_V_1_, A_1_V_2_, A_2_V_0_, A_2_V_1,_ A_2_V_1late_, A_2_V_2_). The visual stimulus was a white disk subtending a visual angle of 1.6° and was presented at 4.1° centrally below the fixation cross, for 10 ms. The auditory stimulus was a 78 dB (SPL) 1000 Hz sine wave tone and was presented for 7 ms with the use of an amplifier (UR22mkII, Steinberg) and a 6.1 cm long, 4 mm wide tube system (ER30, Etymotic Research).

### Data Analysis

The n-back performance was assessed in terms of the accuracy and reaction times (RTs). The accuracy was quantified as the proportion of hits (i.e., correct responses when there was a target letter) minus the misses and false alarms (i.e., responses when there was a non-target letter) over the total number of targets. Regarding the audio-visual stimulation, performance was assessed by estimating, for each combination, the RTs and the proportion of trials when participants reported 0, 1 or 2 perceived flashes.

Previous studies have shown that there is considerable inter-individual variability regarding the perception of the SIFI^71,72^. For the purposes of our study, we focused on subjects that reliably perceived the illusion. Therefore, 8 subjects that didn’t perceive the illusion during the critical A_2_V_1_ trials (i.e., they perceived “2-flashes” in less than 10% or more than 90% of trials^59^) in at least 2 of the 4 conditions (no-back, 0-back, 1-back, 2-back) were excluded from the analysis. Additionally, 6 participants were excluded from the analysis as they markedly failed in perceiving the 2-flashes in the control condition A_0_V_2_ (“2-flashes” response in less than 60% of trials in the “no-back” blocks). In total, 14 subjects were excluded from the analysis. The final sample size (N = 16) is relatively small and limits the external validity of the current findings, however previous SIFI studies showed robust effects using small sample sizes (N = 8 in studies by Shams and colleagues^33,67^).

### Statistical analysis

The statistical significance of the differences in the evaluated parameters (RT and illusion rate) between the different working memory (WM) load conditions was analysed using a repeated-measures analysis of variance (ANOVA). The Mauchly test was used to verify the assumption of sphericity and the Greenhouse-Geisser correction was applied when necessary to correct for non-sphericity. For these cases, the corrected degrees of freedom and *p*-values are reported. Further analysis of the significant effects was performed using post‐hoc paired *t*-tests and the Bayes Factor^73^ (BF) as an indicator of the relative evidence. BFs between 1–3 indicate anecdotal support for the alternative hypothesis (H1) while BF between 3–10 and above 10 indicate respectively moderate and strong support for H1. BF = 1 indicates equal support for H1 and null hypothesis (H0) while BF between 1/3–1, 1/10–1/3 and below 1/10, provide respectively anecdotal, moderate and strong support for H0^74^.

If the data were not normally distributed (failing the Lilliefors test for normality of distribution at alpha level 0.05) we used the Friedman test – a non-parametric alternative to one-way repeated measures ANOVA –, and post-hoc Wilcoxon signed-rank tests to evaluate differences between conditions. In association with each pairwise Wilcoxon test, we report the effect size (*r*; *r* = *Z / n*^*1/2*^, *Z* = Wilcoxon Z-value*, n* = number of observations).

The Holm-Bonferroni correction^75^ was applied for the all the post-hoc pairwise comparisons. An alpha level of 0.05 is used for all statistical tests.

### Data availability

The datasets analysed during the current study are available from the corresponding author on reasonable request.
